# Advances in Biopolymeric Nanopesticides: A New Eco-Friendly/Eco-Protective Perspective in Precision Agriculture

**DOI:** 10.3390/nano12223964

**Published:** 2022-11-10

**Authors:** Ravinder Kumar, Naresh Kumar, Vishnu D. Rajput, Saglara Mandzhieva, Tatiana Minkina, Baljeet Singh Saharan, Dharmender Kumar, Pardeep Kumar Sadh, Joginder Singh Duhan

**Affiliations:** 1 Department of Biotechnology, Chaudhary Devi Lal University, Sirsa 125055, India; 2 Regional Forensic Science Laboratory, Mandi 175002, India; 3 Academy of Biology, and Biotechnology, Southern Federal University, 344090 Rostov-on-Don, Russia; 4 Department of Microbiology, CCS Haryana Agricultural University, Hisar 125004, India; 5 Department of Biotechnology, DRCUS&T, Murthal 131039, India

**Keywords:** biopolymers, nanopesticides, toxicity, release behavior, eco-friendly

## Abstract

Pesticides are essential to contemporary agriculture and are required to safeguard plants from hazardous pests, diseases, and weeds. In addition to harming the environment, overusing these pesticides causes pests to become resistant over time. Alternative methods and agrochemicals are therefore required to combat resistance. A potential solution to pesticide resistance and other issues may be found in nanotechnology. Due to their small size, high surface-area-to-volume ratio, and ability to offer novel crop protection techniques, nanoformulations, primarily biopolymer-based ones, can address specific agricultural concerns. Several biopolymers can be employed to load pesticides, including starch, cellulose, chitosan, pectin, agar, and alginate. Other biopolymeric nanomaterials can load pesticides for targeted delivery, including gums, carrageenan, galactomannans, and tamarind seed polysaccharide (TSP). Aside from presenting other benefits, such as reduced toxicity, increased stability/shelf life, and improved pesticide solubility, biopolymeric systems are also cost-effective; readily available; biocompatible; biodegradable; and biosafe (i.e., releasing associated active compounds gradually, without endangering the environment) and have a low carbon footprint. Additionally, biopolymeric nanoformulations support plant growth while improving soil aeration and microbial activity, which may favor the environment. The present review provides a thorough analysis of the toxicity and release behavior of biopolymeric nanopesticides for targeted delivery in precision crop protection.

## 1. Introduction

Agriculture is the backbone of any economy, and food is a vital resource for living beings. Crops face many issues, including insects, pests, diseases, pesticides, and the toxicity associated with these agrochemicals. Pests cause a total loss of 50% in wheat and 80% in cotton worldwide. Other crops that experience considerable output losses include soybeans (26–29%), maize (31%), rice (37%), and potatoes (40%) [1]. Pest insects account for about 30% of crop losses [2], and weeds also cause substantial losses of about 34%. Pesticides have been used to combat pests; however, the widespread application of pesticides has had a devastating effect on humans and other living organisms, with an increasing incidence of human poisoning. In addition to pests, fungal diseases also affect crops worldwide. In the 19th century in Ireland, a fungal disease destroyed the potato crops to such an extent that it was considered one of the greatest European famines. Likewise, an annual loss of about USD 60 billion in the five most important food crops (wheat, rice, maize, soybean, and potato) as a result of fungi has been reported. Thus, it is important to control fungal diseases in these crops. Enough food to feed about 600 million people each year could be saved by effectively controlling fungal diseases alone [3,4].

In order to expand production to feed the increasing population, the excessive usage of chemical fertilizers and pesticides was carried out during the green revolution of the late 20th century in India, which caused a great loss in soil biodiversity and soil health. The overuse of pesticides has also led to the development of resistance among pests, a major agricultural problem [5]. Nanotechnology can be used to tackle such problems. Active ingredients in conventional formulations are generally mixed with inert materials. However, immediate release leads to the quick loss of these chemicals in the field by various degradation processes, which causes leaching, evaporation, and volatilization. As a result, the active ingredient concentration declines to the minimum level required to maintain biological efficacy. Hence, these conventional pesticides must be applied again and again, which causes soil, human health, and environmental problems. However, this adds chemicals to the soil that persist for a long time or do not degrade easily, deteriorating the soil’s health. Later on, polymeric nanoformulations were developed, which achieved the slow, controlled, and sustained release of active ingredients with less impact on the environment [6]. Biological entities such as bacteria, fungi, actinomycetes, viruses, diatoms, and higher plants are used for environmentally friendly, greener, and safer methods of synthesizing nanoparticles (NPs) that directly inhibit the growth of pathogens [7]. The authors of [8] applied microwave-assisted synthesis to produce silver (Ag) NPs using the hybrid citrus fruit “kinnow” and found them effective against the early blight of tomatoes. Similarly, the authors of [9] managed chickpea (*Cicer arietinum*) wilting disease in vivo by synthesizing silver NPs from the rhizospheric microflora of chickpeas.

Recently, biopolymers as nanoparticulate materials have attracted attention due to their eco-friendly nature and ability to offer the persistent release of the associated active components of pesticides. Natural polymers and gums are biocompatible, sustainable, biodegradable, economical, and non-toxic and are considered an alternative source of raw materials. These natural gums (guar gum and gum acacia) and biopolymers (starch, cellulose, pectin, galactomannan, and lignin) occur in seaweed extracts (carrageenan); fungi (chitosan and chitin); bacteria (polyhydroxybutyrate, xanthan gum); algae (agar, alginate); and plant seeds and stems and their exudates [10,11,12]. The largest portion of biopolymeric materials are derived from plants and animals. The application of NP synthesis based on biopolymers can solve the specific agricultural problem of plant–pathogen interactions, thus presenting a means of crop protection [13,14]. NPs have great potential as ‘magic bullets’ that can be loaded with herbicides, weedicides, fungicides, and fertilizers for the targeted delivery of these agrochemicals to crops. Biopolymeric NPs can release the desired active components at a prescribed rate with a constant dose to the target pest and thus are more effective. Developing controlled-release formulations (CRFs) is highly desirable from the perspective of compliance with international environmental and biodiversity laws. The use of polymers for sustained release started in the early 1970s, due to the higher efficacy of their encapsulated components compared to commercial formulations. To upgrade the value of traditional pesticides, many natural biopolymers such as carrageenan, galactomannans, chitosan, alginate, pectin, cellulose, gum acacia, guar gum, cashew gum, chitin, agar, tamarind seed polysaccharide (TSP), and starch are processed into nanomaterials for the slow and targeted delivery of agrochemicals [15,16,17,18,19,20].

Biopolymeric nanopesticides have various benefits compared to their bulk counterparts, which can be summarized as follows: they cause less environmental contamination due to their low application doses and reduced leaching losses and are thus safe for non-target organisms; they present better efficacy due to their controlled release; and they are resistant to photo-degradation, thus achieving the maximum impact on their target organisms [21]. In addition to the above, biopolymeric nanopesticides are biocompatible, biodegradable, and biosafe, with minimal adverse effects on non-target organisms. They can be degraded easily to form compost, which supports plant growth and performance. Biopolymers increase soil permeability and aeration, ultimately increasing soil microorganism activity. All these properties make biopolymeric nanoformulations the prime choice for encapsulating active components. Their efficacy against different target organisms has been confirmed in many greenhouse and field studies [14,15,22]. The economic cost of biopolymeric nanoparticles and their carbon footprint are also low due to their small size, high surface-area-to-volume ratio, and a very low dose (of both biopolymer and encapsulated pesticide) requirements as compared to their bulk counterparts. Thus, keeping in mind this knowledge gap and their low dose requirements, this review presents a comprehensive overview of biopolymeric NPs as carriers of insecticides, fungicides, herbicides, molluscicides, and acaricides for plant disease management, taking into account their lower toxicity and release behavior.

## 2. Pesticides

Until the end of the 15th century, several toxic chemicals such as arsenic, mercury and lead were used to kill pests threatening staple agricultural crops because of a lack of domain knowledge and the scarcity of resources. In the 17th century, nicotine sulfate was isolated from tobacco leaves and used for the first time as an insecticide. Later, in the 19th century, two more natural pesticides, pyrethrum (obtained from chrysanthemums) and rotenone (extracted from tropical vegetable roots), were introduced [23]. Paul Muller discovered DDT, the first synthetic pesticide, in the 1940s. Subsequently, the growth of synthetic pesticides accelerated with the discovery of BHC, aldrin, 2,4-D, endrin, chlordane, parathion, and dieldrin. All of these pesticides were effective, inexpensive, and used by people all over the world. DDT emerged as the most popular, due to its broad-spectrum activity [24].

Generally, insecticides are grouped into two classes, i.e., systemic or contact insecticides. Based on the physiological functions affected, they can be further classified into different chemical groups. Additionally, to prevent pesticide resistance, modes of action (MoA) must be used in rotation [25]. Fungicides function through contact or penetration. Because systemic mobility throughout a plant is rare, fungicide spraying is quite significant. There are 14 separate MoA groups and 49 Fungicide Resistance Action Committee (FRAC) codes for fungicides [26]. Selective or non-selective herbicides can be sprayed on weeds before they sprout or emerge. Rotation within these groups is necessary to avoid pesticide resistance.

The worldwide human population Is increasing at an annual rate of 1.2%, i.e., approximately 77 million people are added yearly to the existing inhabitants. Asian countries such as China, India, Pakistan, Indonesia, and Bangladesh, as well as Nigeria, are responsible for half of this global annual increment. By 2050, the planet’s population will be around 9.1 billion [27]. Humans and other animals need daily food to maintain their metabolic rate and survival. Cereal grain production worldwide is estimated to total approximately 3300 metric tons by 2050 [28], 60% more than today. Since 1960, noteworthy progress has been made toward improving the nutritional value and ensuring the security of food for living beings. Gross agricultural production has increased more rapidly than the world population, evidenced by the increased availability of food per capita. However, the gap between the quantity of food produced and the global population needing to be feed is likely to increase until the year 2050 [29]. Thus, the need to increase cereal production is inevitable and could be met by disease and pest control methods based on modern nanotechnology in addition to the modern molecular biology methods of increasing the productivity of staple crops.

## 3. Food Loss Due to Insects, Pests, and Diseases

Today, crop growers are facing numerous challenges. The yield and productivity of crops grown for human consumption are in danger because of the prevalence of pests, especially weeds, animal pests, pathogens, and insects. These organisms account for a significant portion crop loss that may be avoided or diminished by modern crop protection measures. Worldwide, USD 2000 billion in economic loss per year has been observed in food production, caused by plant disease (13%), insects (14%), and weeds (3%) [30]. Fungi are responsible for about 70% of diseases in all major crops [31], such as wheat, potato, cotton, tomato, groundnut, cotton, and grapevine [32]. The gravity of the situation can be understood by the fact that in the agricultural industry, annual crop losses due to fungal diseases in the field and after harvest exceed USD 200 billion. In contrast, the USA spends over USD 600 million annually on fungicides [33]. Globally, approximately one fourth of food crops are damaged by fungal toxins such as aflatoxins, ergot toxins, *Fusarium* toxins, patulin, and tenuazonic acid. Worldwide losses due to pests vary from approximately 50% in wheat to more than 80% in cotton production. For other crops, the estimated losses are 26–29% for soybean and 31, 37, and 40% for maize, rice, and tomatoes, respectively [34].

## 4. Biopolymers as Nanoparticulate Materials

The European Commission defined a NPs as “a natural, incidental or manufactured material containing particles, in an unbound state or as an aggregate where, for 50% or more of the particles in the number size distribution, one or more external dimensions is in size range 1 nm–100 nm” [35]. Biopolymers synthesized from naturally occurring sources have several advantageous properties (e.g., biodegradability, biocompatibility, ready availability, and inexpensiveness), which make these biopolymers suitable for numerous research-based applications. The largest portion of biopolymeric materials are derived from plants, such as gum acacia [36], galactomannan [37], pectin, lignin [38], starch [39], and cellulose [40]; animals, such as chitosan and chitin [41,42]; and algae, such as agar [43], alginates [44], and xanthan gum [41]. The polysaccharide cashew gum (CG), a plant exudate, is produced due to the plant’s defense mechanisms against stress. This gum production takes place in all parts of the tree and depends on the maturity of the tree and the environmental conditions. It is partially or sparsely soluble in water and swells, producing a highly viscous solution. It is made of a branched framework of D-galactose units and D-glucuronic acid, L-arabinose, and L-rhamnose. Numerous applications of modified CG have been described. It can be used as an alternative for liquid glue in the paper industry, in the cosmetic industry, as an edible coating for application on apples [45] and mangoes [46], and as an agglutinant for capsules and pills in the pharma sector [47]. It can also be a protective, edible coating on fruits and vegetables. For example, the authors of [48] described CG and carboxymethylcellulose-based formulations that could be used as protective edible coatings on whole red guavas and fruits cut by birds to enhance their shelf life and defense mechanisms. CG is used in many industries.

Chitosan is a non-toxic, biodegradable polymer. Chitin is the primary structural element of crustaceans and some fungal cell walls. It keeps the immune system of plants functioning, secretes enzymes, and enhances plants’ ability to withstand illnesses and insects. The authors of [49] formulated nisin-loaded chitosan/carrageenan nanocapsules using an ionic complexation method and tested their antibacterial efficacy. The concentration of polymer and surfactant affected the particle size and encapsulation efficiency. The release study conducted in vitro indicated slow and sustained release, while the assessment of the antibacterial activity against *Micrococcus luteus* (MTCC 1809), *Pseudomonas aeruginosa* (MTCC 424), *Salmonella enteric* (MTCC 1253), and *Enterobactor aerogenes* (MTCC 2823) indicated that the encapsulated nanocapsules exhibited a better antibacterial effect on the microbes both in vitro and in vivo for prolonged periods of six months, contrary to the components evaluated separately [49].

Similarly, using this biopolymer (chitosan), the authors of [11] synthesized acetamiprid-loaded controlled-release nanocapsules by polyelectrolyte complexation with another natural polymer, sodium alginate. As observed by TEM, the zeta potential results revealed that the nanocapsules formed were stable with a spherical shape. The encapsulation efficiency was 62%, as computed by ultra-high-pressure liquid chromatography (UHPLC). The in vitro release experiment showed maximum release at pH 10.0, followed by pH 7.0 and 4.0, respectively, with a non-Fickian release pattern that was more effective than that of a commercial formulation in soil. The formulation of these nanocapsules could help to cut down the frequency and dose of pesticides by controlling the release and subsequent leaching side effects of conventional pesticides.

Similarly, the authors of [50] prepared chitosan NPs functionalized with β-cyclodextrin and containing carvacrol and linalool. High encapsulation efficiencies (<90%) were shown for both carvacrol and linalool. The synthesized NPs demonstrated acaricidal, repellency, and anti-oviposition activity against mites (*Tetranychus urticae*). The nanoforms were efficient in their acaricidal and anti-oviposition activity, while the unencapsulated compounds showed improved repellency. Table 1 shows examples of biopolymers used as nanoparticulate materials, their characteristics, and their uses in agriculture and other fields.

## 5. Biopolymeric Nanopesticides

The term “nanopesticides” is used to describe any pesticide formulation that “involves either very small particles of an active pesticide ingredient or other small engineered structures with useful pesticidal properties” [70,71]. Nano-formulations for pesticide products offer several benefits, such as increased solubility, kinesis, and durability; significantly fewer active ingredients; less harm caused to non-target organisms, thereby reducing the development of resistance; and protection against premature degradation [72,73].

**Figure 1:**
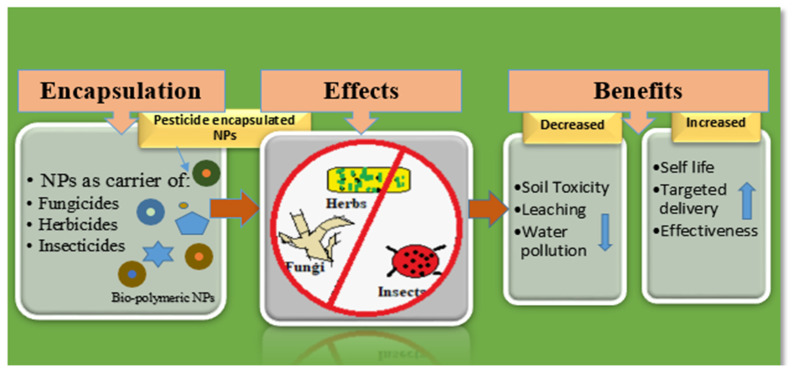
Biopolymeric NPs as carriers of herbicides, fungicides, and insecticides and their benefits.

Nowadays, polymers have become the main components of NP and nanocapsule synthesis, being firmly held together by covalent bonds that make the polymer steadier and more robust than liposomes [74]. Their sizes, morphology, and shapes can be managed more proficiently using polymers by varying their concentration and pH, making them more effective against chemical compounds or metals. Additionally, polymeric nanocapsules can easily be made operational with different substances, increasing their value for achieving certain goals. Natural biopolymers such as chitosan and chitin derivatives are popular objects of research in the field of nanocapsules containing natural polymers [75]. These polysaccharides are biodegradable, made up of linear β-1, 4 linkages of glucose residues, and are the second most common in nature after cellulose. Chitosan can be used to encapsulate and deliver drugs by transforming them into thin membranous films and thus can be used in the biomedical or pharma industry. The application of their mucoadhesive properties in drug transport across cellular membranes supports their possible use in the agrochemical industry [76]. In agriculture, chitosan is well-known for its antimicrobial properties against various microorganisms. In addition to producing defense responses in plants, it also shows antibacterial and insecticidal activity [77,78]. Experimentation on NPs as antifungal agents has been reported by many researchers [79,80]. Natural polymers have been used to encapsulate conventional fungicides to produce nanofungicides [81], nanoinsecticides [82], or nanoherbicides [83] for the slow release of active ingredients and for the enhancement of their disease-prevention efficacy. Figure 1 summarizes the use of biopolymeric NPs as carriers of fungicides, insecticides, and herbicides.

### 5.1. Biopolymeric Nanofungicides

About 20–40% of crop products are lost annually because of pests and pathogens. Traditionally, plant disease management mainly relies on toxic pesticides that are potentially dangerous to the environment and humans. Nanotechnology offers benefits such as reduced environmental and cell toxicity, increased shelf life, and the improved solubility of water-soluble synthetic pesticides. All these factors could have positive impacts on the environment [84]. Fungicides are mainly used to control the diseases caused by fungi in the agricultural industry, which reduce the quality and quantity of produce. The constant use of fungicides is particularly harmful to the ecosystem. Residual fungicides in soil and produce have detrimental effects on the soil properties, microbial community, and animal and human health [11]. Thus, there is a need to find other ways to increase the efficacy and reduce the dose of fungicides. Using nano-encapsulated pesticides reduces their phytotoxicity and improves their diffusion through the cuticle and the sustained release of their active components in the target site. Using biocompatible polymers for the nano-entrapment of agrochemicals can further reduce toxicity by achieving the targeted and controlled release of the fungicides [10]. Nowadays, there is an increasing interest in non-toxic and biocompatible polymers such as chitosan, carboxymethyl, sodium alginate, starch, cellulose, gelatin, and pectin [85].

Different amounts of the low-water-soluble fungicide pyraclostrobin were added to chitosan–lactide copolymer NPs. Three days post-application, it was discovered that the nanofungicide was either equally as effective as commercial pyraclostrobin or less effective in preventing *C. gossypii* inhibition. However, compared to acting alone, an increase in inhibition was observed five days after application [86]. Similarly, another research group [12] employed polymeric and solid lipid NPs (SLNPs) for the persistent release of the antifungal drugs tebuconazole and carbendazim, which are common fungicides. Compared to the SLNPs, these polymeric NPs released around 47% of the fungicides over six days. The high association efficiency (>99%) of both formulations demonstrated a strong ionic interaction. When the effect of commercial and NP formulations on plant emergence was assessed using *P. vulgaris* seeds, the germination index of the seeds (92%) was not modified by either formulation (with or without fungicides). 

In another trial, kaempferol (another low-soluble fungicide) loaded onto lecithin/chitosan displayed 67% inhibition efficacy after 60 days against *Fusarium oxysporum* infected petri dish [87]. The effect of carbendazim-loaded biopolymeric NPs of chitosan and pectin compared to carbendazim alone on *Aspergillus parasiticus* and *Fusarium oxysporum*, the authors of [88] discovered an enhanced fungal inhibition rate. According to phytotoxicity studies, maize, cucumber, and tomato seeds did not experience any negative effects from the nanoformulated carbendazim when planted and allowed to grow roots. Similarly, the authors of [9] incorporated copper-oxy-chloride into chitosan–CuO/ZnO nanoformulations and managed *Fusarium oxysporum* f. sp.ciceri (FOC) in chickpeas. Similar process was used by the authors of [89] to create chitosan NPs (CNPs) from chitosan (CS). The phytopathogenic fungi *Phytophthora capsici, Colletotrichum gelosporidies, Fusarium oxysporum, Sclerotinia sclerotiorum,* and *Gibberella fujikuori* were used as test subjects to assess the NPs’ in vitro antimicrobial activity. Following closely behind *P. capsici*, the CNPs revealed the greatest growth-inhibitory effects on *F. oxysporum* mycelial growth. Their antibacterial activity against *Xanthomonas* and *Erwinia* bacterial strains were also tested. The study showed that both CS and CNPs have tremendous potential as nanofungicides, as they markedly inhibited the growth of fungal and bacterial strains in tomatoes and could be used as crop protectants. Similarly, the authors of [90] found that the encapsulation of the fungicide spinosad in chitosan–isoleucine NPs protected the fungicide against photo-degradation, thereby increasing the effectiveness of this commercial fungicide. Likewise, the authors of [91] demonstrated a 74.5% reduction in basal stem rot disease caused by *G. boninense* in palm trees by applying chitosan hexaconazole-dazomet. When encapsulated in lignin NPs, the fungicides azoxystrobin, pyraclostrobin, tebuconazole, and boscalid azoxystrobin inhibited the growth of test fungi (*Phaeomoniella chlamydospora* and *Phaeoacremonium minimum)* under in vitro conditions after 96 h. These fungi cause grapevine trunk disease [92]. Table 2 lists some biopolymeric nanoformulations that have been used as nanofungicides.

### 5.2. Biopolymeric Nanoinsecticides

Insects represent the largest populations of animals and are found in all possible environments throughout the globe. Several evolutionary aspects are responsible for their success, including their habit diversification, reproductive potential, desiccation-resistant eggs, ability to metamorphosize, and development of wings and exoskeletons. Approximately 500,000 species of identified insects feed on green leaves. The majority of them eat from a small range of plant species, and some of them are even species-specific [108]. These insects are tiny organisms that attack plants en masse and contribute significantly to crop losses, mainly during the period of crop flowering. They mainly feed on crop leaves, flowers, and fruits, making them unusable. Some insects also damage fruits post-harvest. The authors of [10] synthesized sodium alginate nanocapsules loaded with the insecticide imidacloprid (IMI) by the water-in-oil-in-water (W/O/W) double-emulsion method. An aqueous solution of sodium alginate was supplemented with a solid form of IMI and then sonicated and emulsified by methylene chloride and dioctyl sodium sulfosuccinate. The formed NPs were harvested by ultracentrifugation and tested on a leafhopper, an insect that consumes plant sap, to evaluate the efficacy of the IMI nanoformulations. Leaf infestation was assessed by counting the leafhopper population on three leaves in a scientific manner. The results showed that blank NPs had no positive effect; however, the nanoencapsulated IMI was more effective than the free insecticide or commercial form of IMI. Toxicity studies also discovered that the nanoformulated IMI was considerably less toxic than the trade pesticide.

Similarly, the authors of [109] encapsulated the hydrophilic carbamate insecticide methomyl in polymers. This is a broad-spectrum insecticide, but when applied via water it undergoes rapid decomposition in the air or sunlight, so nanoencapsulation is vital to avoid its premature degradation. An amphiphilic, photo-crosslinkable carboxymethyl-chitosan (Az-CMCS) biopolymer was formulated by reacting a previously prepared ethanolic solution of azidobenzaldehyde (Az) with an aqueous solution of carboxymethyl chitosan (CMCS). Methomyl was dissolved in an aqueous solution of Az-CMCS at pH 4.0 under sonication. Under these conditions, the amphiphilic polymer self-assembled into methomyl-loaded nanocapsules with an aqueous core, resulting in a more stable and slow-releasing nanoinsecticide with premature-degradation-resistant properties.

Similarly, *Helicoverpa armigera* larvae were managed using carboxymethyl chitosan and sodium alginate nanoformulations with methomyl and pyridalyl insecticides [110]. The low-water-solubility insecticide acetamiprid was transformed into a controlled-release nanoformulation [11]. In order to create acetamiprid-loaded alginate–chitosan nanocapsules, ionic pregelation and polyelectrolyte complexation were used. A polyionic complex was created with a loading efficiency of 62% due to ionic interactions between the positively charged ammonium groups of chitosan and the negatively charged carboxylate groups of alginate. Studies on its controlled release in various soil mediums showed that it was superior to commercial acetamiprid. Acidic soils achieved the best results. Essential oils have also been encapsulated in chitosan and other natural biopolymers to increase the insecticidal activity of biopolymers. The authors of [50,111,112,113] synthesized chitosan/gum arabic, chitosan/zinc oxide, and chitosan NPs encapsulating geraniol, azadirachtin, carvacrol, and linalool to control whitefly (*B. tabaei*), groundnut bruchid (*C. serratus*), and mite (*T. urticae*), respectively. The authors of [17] formed spinosad- and permethrin-loaded chitosan NPs to control *Drosophila melanogaster.* The insecticides emamectin benzoate and thiamethoxam were encapsulated into carboxymethyl chitosan and cellulose NPs to control the insect pests *Mythimna separate* and *Phenacoccus solenopsis* by the authors of [20,113], respectively. In a comprehensive review, the authors of [114] described the use of 15 polymers as nanocarriers of insecticides. Table 3 summarizes some of the biopolymeric NPs that have been tested as carriers of insecticides and active compounds.

### 5.3. Biopolymeric Nanoherbicides/Nanoweedicides

Herbs, or weeds, are unwanted plants that grow alongside cultivated crops. Herbs are becoming a new challenge for modern agriculture. With the advent of modern agrochemicals, agricultural productivity and disease resistance have increased. This has also produced new types of herbs that must be eradicated to improve productivity and soil maintenance. 

The herbicide paraquat is a fast-performing, non-selective, and widely used contact herbicide. However, its water solubility and soil sorption can cause toxicity problems in non-target organisms and humans via groundwater and rivers. To combat this toxicity problem, 635 ± 12 nm alginate/chitosan conjugated NPs were prepared as a green carrier system for the herbicide paraquat [134] with a zeta potential of −22.8 ± 2.3 mV and association efficiency of 74.2%. The conjugated NPs with encapsulated paraquat improved the release profile of the herbicide and its interaction with the soil, demonstrating that this formulation can effectively reduce the adverse impacts of paraquat. Grillo and others [135] performed similar work with paraquat-loaded chitosan/tripolyphosphate (CSTPP) NPs. An encapsulation efficiency of 62.66 ± 0.77% was achieved, representing good affinity between the CSTPP NPs and the active component of the herbicide. The herbicidal activity was investigated in maize (*Zea mays*) and mustard (*Brassica* sp.). Both free and encapsulated forms of paraquat caused limp leaf necrosis within 48 h, a distinctive effect of this herbicide on both plants. In *Z. mays*, the use of the nanoherbicide caused more significant necrosis, possibly due to the better adhesion of the NPs to the leaf, as NPs have an increased surface-area-to-volume ratio. It is likely that the greater sensitivity of the plants to the herbicide had a more negligible effect in *Brassica*. NPs increase particle adhesion to surfaces due to their increased surface area, enhancing the effectiveness of the associated active agents.

In comparison to the commercial herbicide MTT, testing revealed that nanoherbicides had reduced cytotoxicity to Chinese hamster ovary (CHO) cells, whereas nanoparaquat induced modest chromosome damage in *A. cepa* [135]. The authors of [19] embedded paraquat in pectin, chitosan, and sodium tripolyphosphate (PEC/CS/TPP) NPs for sustained pesticide release. Alveolar and oral cell lines were less hazardous to the encapsulated herbicide. The nanoformulation mutagenicity of a model using the Salmonella typhimurium strain was markedly lower than that of paraquat in its commercial forms. The soil sorption of paraquat and the deep soil penetration of the NP-linked herbicides decreased. After encapsulation, paraquat’s herbicidal efficacy against maize and mustard was sustained and substantially improved. The authors of [83] assessed the herbicidal effect of glyphosate in chitosan nanoformulations on three weed species: gallant soldier (*G. parviflora* Cav), white goosefoot (*C. album* L.), and common sorrel *(R. acetosa* L.), among the most glyphosate-resistant weeds. The advantage of the above formulation was that chitosan dissolved in a water solution of glyphosate may play a double role as an environmentally friendly adjuvant (sticker) and as a biopolymeric carrier for the prolonged release of glyphosate. Some of the biopolymeric NPs that have been used as carriers of herbicides are listed in Table 4.

### 5.4. Nanonematicides

Nematodes are common soilborne organisms, and more than 4100 plant-parasitic species cause significant damage to crops in sandy soil and water-stressed conditions, representing approximately USD 80–118 billion in losses worldwide [147,148,149]. Nematodes mainly feed on roots and can lead to the entry of other disease-causing microbes, such as bacteria and fungi, further worsening crop damage. Conventional nematicides have been found to be environmental toxins, and new approaches in the form of NP applications are being explored. The microemulsion polymerization process was used to create nanocapsules of lansiumamide B (N-methyl-N-cis-styrylcinnamamide) with nematicidal activity against *Bursaphelenehus xylophilus* and *Meloidogyne incognita* (LC_50_ values of 2.14 and 19.36 mg/L, respectively, after 24 h). Additionally, treatment with nanocapsules, regular polymers, and ethoprophos (an insecticide and nematicide) saw 68.42, 36.84, and 26.32% reductions in the disease’s progression, respectively. The average number of root knots in *Ipomoea aquatica* decreased by 83.94, 78.03, and 63.66%, respectively, showing that the nematicide nanoformulation worked more effectively and maintained its effectiveness against plant parasitic nematodes for a longer period [150].

The effectiveness of starch-stabilized silver NPs against the root-knot nematode *M. incognita* was investigated in [151]. Under in vitro conditions, this nanoformulation inactivated > 99% of the root nematodes in 6 h. The population of nematodes in the soil samples treated with this nanoformulation (150 g/mL) in the in vivo experiment was reduced by 92 and 82% after 4 and 2 days of exposure, respectively, compared to the nontreated soil samples. In a related study, the authors of [152] discovered that administering silver NPs (AgNPs) with particle sizes of 200, 400, and 800 mg/mL to *M. incognita* resulted in 100% immobility and mortality, with a determined LC_50_ value of 100 mg/mL. The same nanoformulation at 0.02, 0.01, 0.005, 0.0025, 0.00125, and 0.0007% concentrations (*w*/*w*) controlled *M. incognita*; however, treatment with 0.02, 0.01, and 0.005% AgNPs was lethal to tomato plants and reduced tomato root and stem length and fresh weight significantly in comparison with the control. The authors of [153] applied the flash nanoprecipitation technique to produce abamectin-loaded NPs with a loading capacity > 40% and encapsulation efficiency > 95% using the amphiphilic copolymers poly(lactic-co-glycolic acid)-b-poly (ethylene glycol) (PLGA-b-PEG), poly(d,l-lactide)-b-poly(ethylene glycol) (PLA-b-PEG), and poly(caprolactone)-b-poly(ethylene glycol) (PCL-b-PEG); these were tested against the southern root-knot nematode *Meloidogyne incognita.* There are no reports regarding the use of biopolymers as carriers of nematicides. Table 5 lists some of the polymeric nanonematicides that have been developed.

### 5.5. Nanomolluscicides and Nanomiticides

Mollusks are soft-bodied animals. They also cause significant damage to the crops in many regions of the world, besides being the reason for many livestock and human diseases. Table 6 summarizes several of the studies that have been performed using polymeric NPs to target mollusks.

Similarly, metallic nanocomposites have been used extensively to control termites in woody species. However, little work has been carried out using polymeric NPs for this purpose, as shown in Table 7.

## 6. Antimicrobial Activity of Biopolymeric Nanoparticles Alone

NPs have been studied comprehensively in regard to their antimicrobial properties to determine their efficacy against fungal and bacterial diseases in plants and animals. The efficacy of NPs against antibiotic-resistant strains of bacteria and fungi lies in their small size. On the nanoscale, particles act as molecules when interacting with a cell, allowing them to penetrate the cell membrane easily and positively affecting the central molecular pathways. Their high surface-area-to-volume ratio increases contact with the target, making NPs a magic bullet for drug delivery. They may be synthesized from biological entities such as bacteria, fungi, biopolymers, and lipids, making them useful in various fields. NPs’ interactions with fungal pathogens rely on the size and shape of the NPs; thus, disease control and toxicity are governed by these factors.

Several experiments have been performed to determine the antimicrobial activity of NPs. The authors of [157] prepared chitosan NPs from diverse concentrations of low-molecular-weight (LMW) and high-molecular-weight (HMW) chitosan. These were found to show superior inhibitory activity, i.e., a low MIC (minimum inhibitory concentration) for *F. solani* (MICLMW = 0.86–1.2 mg/mL and MICHMW = 0.5–1.2 mg/mL) and *C. albicans* (MICLMW = 0.25–0.86 mg/mL and MICHMW = 0.6–1.0 mg/mL) compared to the solution form (MIC = 3 mg/mL for both molecular weights and fungal strains). The particle size and zeta potential of the chitosan NPs also impacted their inhibitory effect. Additionally, *Aspergillus niger* was unaffected by the chitosan NPs, except for the HMW NPs at higher concentrations. Trimethyl chitosan (TMC) NPs showed insignificant antifungal activity. Therefore, the parent compound, i.e., chitosan, can be used as a natural antifungal agent in its NP form to enhance its antifungal activity.

The authors of [158] used low-molecular-weight chitosan as a reducing and stabilizing agent to create silver NPs between 10 and 15 nm in size. The production of NPs was confirmed by the UV–Vis spectrum, EDS (energy-dispersive X-ray spectroscopy), and FESEM (field emission scanning electron microscopy), and the structure of the composite of chitosan and AgNPs was made clear. The conidial germination of *C. gloeosporioides* was significantly reduced by the chitosan–Ag nanocomposite (treated with 30 mM AgNO_3_) compared to chitosan alone. The chitosan–Ag nanocomposite suppressed conidial germination by 44, 70, and 78% at concentrations of 0.1 (0.00001%), 1.0 (0.0001%), and 10 g/mL (0.001%). The concentration of 100 g/mL (0.01%) hindered spore germination. This effectively reduced the frequency of anthracnose in mango and inhibited the conidial germination of *C. gloeosporioides*. This nanocomposite could find applications in preventing dormant infections of *Colletotrichum* in mango, thus avoiding huge crop losses and promoting the export of high-quality fruits and vegetables.

Fusarium wilt is a fungal disease of chickpea (*Cicer arietinum*) caused by *Fusarium oxysporum f*. sp. *ciceri* (FOC) and has seed- and soil-borne origins. The authors of [9] performed in vitro and in vivo studies of chitosan (CS) and its nanocomposites as antifungal agents against FOC. Chitosan–copper oxide nanocomposites (CS-CuO) and chitosan–zinc oxide nanocomposites (CS-ZnO) were the most effective against various FOC concentrations, i.e., 50, 100, and 200 µg/mL. The CS NPs and chitosan–silver (CS-Ag) nanocomposites were effective and more efficient than standard fungicides, i.e., copper oxy-chloride (CuOCl). Based on the in vitro results, a 100 µg/mL concentration of all nanoformulations was selected for in vivo plant studies under pot conditions. The highest wilt disease reduction (46.67%) was observed in CS-CuO nanoformulations, followed by CS-ZnO (40%). In contrast, CS-Ag and CS caused only a 33.33% reduction in wilt incidence, representing the lowest impact. All synthesized nanoformulations showed excellent antifungal efficacy, inhibited the pathogens, and promoted the growth of the chickpea plants compared to the untreated plants.

The authors of [159] reported on the applications of chitin- and chitosan-based polymers in controlling plant diseases. Similarly, the authors of [160] found that compared to untreated plants, adding a chitosan nanoformulation to the soil marginally increased the growth of tomato plants and decreased the occurrence of *Fusarium* wilt disease. Utilizing chitosan nanoformulations complexed with both proteins and CaCO_3_ in protein/CaCO_3_/chitin nanofibers proved more successful at preventing disease than protein alone or protein/chitin nanofibers. The authors of [161] employed oligochitosansilica/carboxymethyl cellulose NPs to control *Phytophthora infestans* at a concentration of 800 mg/L (the lowest concentration that inhibited fungal growth). The antimicrobial activity of some biopolymeric nanocomposites is summarized in Table 8.

## 7. Release Dynamics of Nanopesticides

Modern pesticide formulations are a burden to farming systems because they build up in the soil and ecosystems and can have detrimental impacts on humans and other living things. Nanotechnology makes it easy to control the release of agrochemicals and deliver certain macromolecules to specific locations for improved plant disease resistance, enhanced plant growth, and effective nutrient use. With reduced contact with the environment, nanoencapsulation provides the advantage of safer handling and more effective pesticide usage, which ensures environmental protection. In plant entomology, nanotechnology focuses on particular agricultural issues in the interactions between plant pests and provides novel crop security strategies. In their study, the authors of [10] delivered the pesticide imidacloprid (admire) to plants in a nanoformulation and examined its impacts. Their findings may offer some guidance for the safe application of this cutting-edge technology to increase crop output and safety.

The authors of [172] developed a nanoparticulate system based on ionic gelation between chitosan and arabic gum for loading insulin. The release profile of insulin in phosphate-buffered solutions (pH 6.5 and pH 7.2) was found to be entirely different from that in an acidic medium (pH 1.2). The increased solubility of chitosan in an acidic medium and the improved swelling of arabic gum chains at pH 6.5 resulted in lower insulin release at pH 6.5 as compared to other pH values. A non-Fickian transport pattern was observed, possibly indicating that release was controlled by the diffusion or relaxation of the biopolymer chains. 

The authors of [173] prepared a spherical-shaped, nisin-loaded tripolymeric nanoformulation using chitosan, sodium alginate, and pluronic F68, with a mean particle size of 208.2 nm. The Fourier transform infrared study did not show any ionic interaction among the components of the composite NPs. Th in vitro release experiment showed an initial burst release in the first week, followed by the sustained release of nisin from the formulation in the second week. This confirmed the experiment’s success and provided favorable prospects for the use of these biopolymers in agriculture. 

**Figure 2:**
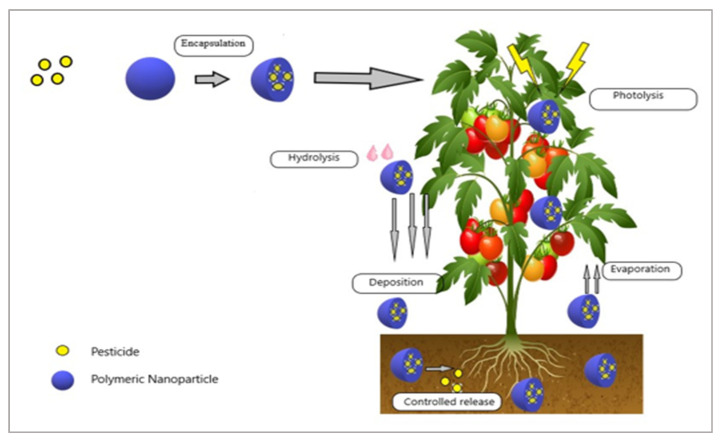
Encapsulation of pesticides into NPs for site-directed delivery and to avoid premature degradation.

Similarly, guar gum was successfully acrylated by the authors of [11] to create guar-gum-grafted polyacrylic acid. The grafted biopolymer displayed enhanced stimulus responsiveness, low viscosity, and improved heat stability. During grafting, the pesticide chlorpyrifos was captured as a model bioactive molecule to examine the effectiveness of its entrapment and release behavior. The sizable release of chlorpyrifos was noticed when a methanolic buffer (pH 7.4, 25:75 *v*/*v*) was utilized. The calibration curve determined that the formulation’s entrapment efficiency was 60%. The goodness-of-fit model-dependent technique and other kinetic models showed that the release was concentration-dependent and followed first-order kinetics for a slow and prolonged period. The site-directed delivery of biopolymeric NPs is represented schematically in Figure 2.

The partial release of carbendazim from chitosan-pectin NPs into the media at various pH values was noted [88]. After 48 h, the average cumulative percent release of carbendazim from the nanoformulation was 61.9 ± 0.1% at pH 4.0, 50.4 ± 0.13% at pH 7.4, and 62.8 ± 0.13% at pH 10.0. In contrast, the percentage release of pure carbendazim was recorded to be 82.4 ± 17% at pH 4.0, 67.7 ± 0.1% at pH 7.4, and 86.8 ± 0.2% at pH 10.0. These data supported the persistent release of NPs, because the active ingredient was contained between the core of the biopolymeric nanoformulation, which shielded it from environmental variables such as light, water, oxygen, and hydrolysis. This release pattern was observed in the experiments. Polybutylene succinate (PBS) and poly lactic acid (PLA) were used together in [174] to improve encapsulation effectiveness and ionic interaction. The resulting 7.2 μm (micrometer) microsphere demonstrated a high degree of encapsulation efficiency. Comparative studies showed that the microsphere had a longer period of sustained release than the difenoconazole-azoxystrobin (5:8, 32.5% *w*/*v*) suspension concentrate due to its complex stoichiometry, which was optimal for refining the disease efficacy of the pesticides. These results verified that such a pesticide microsphere delivery structure could be a promising target for further exploration.

Similarly, the authors of [82] applied sodium alginate, polyacrylamide (PAM), and montmorillonite (MMT) to construct several stretchable double-network nanocomposite hydrogels for controlling the release of λ-cyhalothrin. Adopting sodium alginate with the appropriate incorporation of MMT significantly improved the system’s tensile properties, loading efficiency, and slow-release behavior. The hydrogels’ maximum fracture strain and loading efficiency reached 2000% and 81.30%, respectively. The hydrogel with 5% MMT content showed the least cumulative release percentage of 6.68% over 87 h, and the pesticide release curves fit into the Weibull model.

The authors of [175] applied the oil/water emulsion solvent evaporation technique to polylactic acid and fungicide azoxystrobin and demonstrated that the cumulative release percentage was inversely related to particle size. Another study [81] used ionic gelation to encapsulate commercial hexaconazole in chitosan NPs, resulting in a continuous release of 99.91% over a protracted period of 86 h. Using a similar synthesis process and the same biopolymer but different agricultural chemicals (spinosad and permethrin), the authors of [143] formulated a N-hexanoyl-O-glycol chitosan–atrazine micelle, which enhanced the water solubility of atrazine three-fold. The successful release of dicamba dimethylamine in aqueous and agricultural soils was described by [176] using Cu–chitosan NPs synthesized by a chemical reduction method. The release mechanism was tested using the Korsmeyer–Peppas model (KP) for a calcium-alginate–sulfentrazone (a herbicide) nanoformulation [18]. The high-molecular-weight chitosan biopolymer matrix caused a slower release of the herbicide glyphosate than the low-molecular-weight chitosan system [83]. The time taken for 50% of the herbicide imazethapyr to be released increased from 11.30 days to 43.73 days using nanoformulations of alginate–cellulose synthesized via the ionotropic gelation method [177]. The research on active ingredients encapsulated in biopolymer-based controlled-release matrices and their use in agriculture is summarized in Table 9.

## 8. Toxicity Profile of Nanopesticides

The indirect or direct exposure of humans to pesticides causes several health issues, such as obesity, endocrine disorders [195], cancer [196], and neurological illness [197]. The authors of [198] found organochlorine-pesticide-induced carcinogenic effects in some patients. Similarly, the authors of [199] found organophosphate and organochlorine pesticide exposure in children of farm workers aged 6 to 11 years. The case group’s serum levels of beta-HCH, 4,4 DDE, and 4,4 DDT were considerably higher than those of the control group. 

For nanoparticulate systems, it is not sufficient to characterize the particles and demonstrate their good association with the active compound of the pesticide or other drug. It is also essential to assess their toxicity to target and non-target organisms to further the holistic and sustainable development of the environment. Toxicity profiling is a prerequisite for any drug or agrochemical before it is released on the market. A group of microbiologists [200] studied the influence of nanohexaconazole on soil nitrifiers for 28 days in three replicates with NP doses of 10, 25, and 50 g/ha in 98% humidity at 25 °C. Soil nitrification activity was observed, and it was found that the ammonium, nitrogen, and nitrate-N curve was the same for both the nano- and commercial pesticides. No adverse effects of nanohexaconazole were observed on the two key microorganisms, *Nitrosomonas* and *Nitrobacter* species.

**Figure 3:**
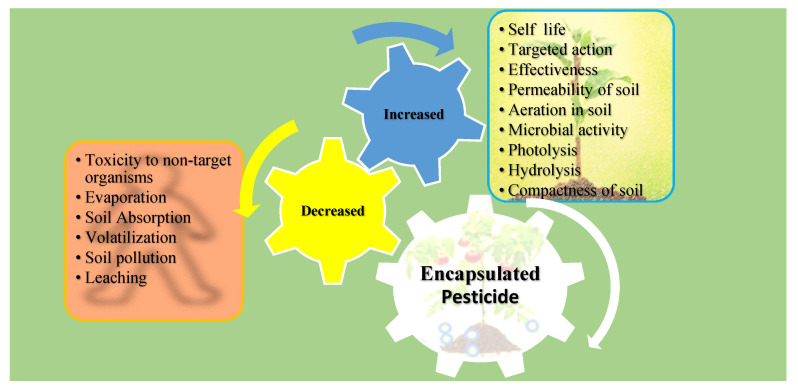
Impacts of pesticides encapsulated in biopolymeric NPs on soil, organisms, and the environment.

Similarly, cytotoxicity and genotoxicity assays of the herbicide paraquat on Chinese hamster ovary (CHO) cells and *A. cepa* seeds were conducted in [136]. The IC_50_ value for free paraquat was about 0.12 mg/mL, while the NPs (with or without paraquat) showed almost no toxicity at the same concentrations tested for 24 h. The genotoxicity data revealed that there was increased DNA damage for all the treatments as compared to the negative control. However, the damage was insignificant when blank NPs were used, as greater damage was observed when free paraquat was used. Figure 3 summarizes the impacts of biopolymeric NP-encapsulated pesticides on the environment.

When carbendazim-loaded biopolymeric NPs were tested against *Aspergillus parasiticus* and *Fusarium oxysporum* and compared to carbendazim alone, the authors of [88] discovered an increase in the fungal inhibition rate. Phytotoxicity studies confirmed that maize, cucumber, and tomato seeds germinated and grew roots more efficiently when carbendazim was nanoformulated. The impact of carbendazim and tebuconazole on mouse fibroblast cells was studied in [12] using polymeric and solid lipid NPs. The cellular viability assays performed on normal cells (3T3 and MC3T3) demonstrated that, regardless of the cell types used, the NPs were less toxic than the commercial fungicides. Cellular viability levels below 25% and 60% at the maximum concentration for the cell lines 3T3 and MC3T3, respectively, indicated the cytotoxicity of the fungicides. The effect of exposing the cells to the commercial formulation was dose-dependent. However, the adenocarcinoma cell (HeLa) results revealed that the fungicide-loaded solid lipid NPs had higher cytotoxicity than the commercial formulations. This could be ascribed to the variation in how well the cells absorbed these NPs. In its commercial and nanoformulation, hexaconazole was more hazardous to Vero cell lines when the pesticide concentration was increased from 10 to 20 ppm [98]. The pesticide-free blank nanoformulation exhibited no cytotoxicity, which could be attributed to the biocompatibility of the polymers used to create the nanocapsules.

In contrast to seeds treated with pure carbendazim, which exhibited a decrease (up to 60%) in germination, seeds treated with nanoformulations had a 96% germination rate, according to a study in 2017 [88]. During the phytotoxicity investigation, a significant decrease in root length and germination % was seen in seeds treated with carbendazim compared to those treated with nanoformulations. The authors of [201] found that the fungicide prochloraz encapsulated in chitosan/silica reduced zebrafish toxicity six-fold compared to commercial prochloraz. In their study, Dong et al. [90] successfully demonstrated that the encapsulation of the fungicide spinosad in chitosan–isoleucine NPs protected the fungicide against photo-degradation.

The authors of [138], in their study on soil biota under the application of the herbicides imazapic (IMC) and imazapyr (IMR) encapsulated in alginate/chitosan (ALG/CS) and chitosan/TPP (CS/TPP) NPs, found that after 30 days, higher numbers of bacteria (compared to the negative control) were observed in the soils treated with the NPs alone (CS/ALG and CS/TPP), while the treatment with CS/ALG/IMC + IMR showed the greatest similarity to the negative control. The *Allium cepa* assay demonstrated that the encapsulation of the herbicides could reduce the extent of damage compared to the free compounds when applied to black-jack (*B. pilosa).* Similarly, the authors of [19] found that the encapsulated herbicide paraquat was less toxic to alveolar and mouth cell lines than its trade form. Additionally, the toxicity of NPs for the A549 cell line was lower than for the KB cell line for most doses. While applying sodium alginate NPs to cucumber plants, the authors of [145] observed that the leaching of the commercial herbicide tebuthiuron in the soil reached 40–50 cm deep, while for the nanoformulations, it reached 20–30 cm deep, suggesting that they were a safe alternative to the commercial herbicide. The authors of [47] assessed the cytotoxicity of metsulfuron-methyl-loaded pectin NPs using healthy cell lines (Vero cell lines) and compared it to that of the commercial herbicide. They also used a pectin nanocarrier to conduct an in-field evaluation using *Chenopodium album* plants. The findings suggested that using herbicide-loaded NPs could minimize the dose requirements of herbicides while improving their effectiveness and environmental safety. Mancozeb-loaded chitosan–gum-acacia NPs at 0.25 mg/mL showed the least cytotoxicity (25.92%), which was considerably lower than the cytotoxicity of commercial mancozeb (33.72%), according to [106]. The cytotoxicity of all three mancozeb-loaded nanoformulations at 2.0 mg/mL was also much lower than that of commercial mancozeb (87.61%), showing values of 61.40, 69.99, and 77.78%, respectively. The very low toxicity of the blank NCs at all concentrations demonstrated that mancozeb, not the nanocomposites, was the cause of the cytotoxicity.

Thus, NPs provide a more environmentally friendly method of battling human infections and fungal crop diseases by reducing the cytotoxicity and genotoxicity of pesticides for non-target species [202,203,204]. The authors of [83], while working to eliminate the deadliest weeds, common sorrel (*R. acetosa* L.) and white goosefoot (*C. album* L.), found that a high concentration of chitosan NPs with encapsulated glyphosate caused a reduction in toxic effects against the roots of *Avena sativa* and *Raphanus sativus.*

## 9. Conclusions, Challenges, and Future Prospects

The application of nanotechnology in agriculture throughout the globe is at its embryonic stage. Modern agrochemicals such as nanopesticides and nanofungicides are being developed for enhanced plant growth, nutrition, and protection against diseases to meet the food demands of the ever-increasing world population. Biopolymeric NPs such as chitosan, carrageenan, guar gum, gum acacia, and sodium alginate efficiently dispense pesticides and nutrients precisely and with high site-specificity. From the perspective of plant–pathogen interactions and the delivery of systemic agrochemicals to specific sites, they provide innovative solutions to problems such as resistance to synthetic pesticides, disease outbreaks, and leaching, thus reducing environmental pollution. Functionalized NPs can pass through a plant’s vascular system and guide agrochemicals (fungicides, herbicides, and insecticides) or other substances (plant hormones, elicitors, and nucleic acids) into targeted, localized areas of plant tissues with sustained release. Natural polymer-mediated NP synthesis provides a benign synthesis route with better control over NP morphology, which ultimately decides the toxicity of the synthesized NPs. Toxicity profiling is essential to achieve sustainable agriculture goals. Biopolymeric NPs are a promising technology, representing a greener approach to target-specific disease control and the sustained release of pesticides in modern agricultural practices without compromising the ecosystem.

Nanotechnology has progressed rapidly in other industries but lags in agriculture. Despite their advantages, many biopolymeric NP-based products have not been commercialized yet for practical implementation in agriculture. This is due to the lack of sufficient field trials, sophisticated labs/instruments, a regulatory framework, and guidelines. Consistent field trials are a prerequisite for the massive adoption or commercialization of nanopesticides, as results under laboratory or greenhouse conditions do not always reflect those in the field. Material scientists and biologists need to understand the fundamental questions related to nanotechnology research and address the research gap to facilitate the production of biocompatible, biodegradable, and safe commercial biopolymeric nanoproducts that can be used without disturbing the ecological balance. Multidisciplinary and collaborative research projects in this field will provide a platform for making plant protection a reality.

## Figures and Tables

**Table 1:** Biopolymers used as nanoparticulate materials in agriculture and other fields: sources, structure, characteristics, and uses.

Biopolymer	Source	Structure	Characteristics	References
Galactomannan	Extracted from the endosperm of leguminous seeds	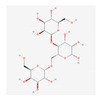 Composed of a linear chain of β-1, 4-D-mannopyranose to which α-1,6-D-galactopyranose units are attached.	Used as films/coatings, gel agents, emulsion stabilizers, and thickeners.	[37,51]
Chitosan	The deacetylation of chitin (found in crustaceans and fungi)	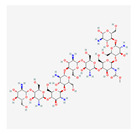 Deacetylated chitin, a linear polysaccharide of deacetylated beta-1, 4-D-glucosamine. Chemical name: (1,4)-2-Amino-2-deoxy- Beta-D-glucan. Functional groups: CH_2_OH, O, OH, NH_2_.	Biocompatible, biodegradable, non-toxic, polyoxysalt formation, film-forming ability, adsorption properties; used in agriculture, food, pharma, and health sectors.	[41,52]
Tamarind seed polysaccharide	From the seed of the Indian date, tamarind (*Tamarind indica* L.)	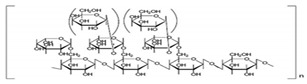 The monomer of three main sugars,2 glucose, galactose, and xylose, in a molar ratio of 3:2:1. Made up of a (1 → 4) β-D-glucan backbone substituted with side chains of α-D-xylopyranose and β-D-galactopyranosy linked (1 → 2)-α-D-xylo-pyranose linked (1 → 6) to glucose residues. Functional groups: CH_2_OH, O, OH.	High viscosity, adhesivity, non-carcinogenicity, broad pH tolerance, and biocompatibility.	[53,54]
Starch	Major carbohydrate form that is held in seeds, roots, rhizomes, and tubers	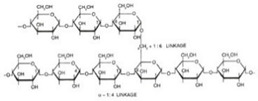 Chemical formula: (C_6_H_10_O_5_)n. Amylopectin (α-amylose) and amylose (β-amylose) constitute starch. Amylopectin (α-amylose) is branched, while β-amylose consists of linear chains.	It is a tasteless, odorless powder insoluble in alcohol or cold water. Native starch’s significant swelling and quick enzymatic breakdown make it unsuitable for controlled-release medication delivery systems.	[55,56]
Cellulose	A structural element of the major cell walls of oomycetes, several types of algae, and green plants	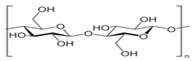 Cellulose (C_6_H_10_O_5_)n consists of a linear chain of several β(1 → 4) linked D-glucose units. Function group: OH, O.	This non-toxic polymer, with high tensile and compressive strength, is utilized in the food, cosmetics, pharmaceutical, and nanotechnology industries.	[40]
Chitin	The primary sources of chitin are crustacean shells; also found in mycelia and the spores of many fungi	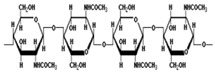 Poly β-(1 → 4)-N-acetyl-D-glucosamine, i.e., N-acetyl-d-glucosamine units with β-(1,4) bonds. Functional groups: CH_2_OH, O, OH.	Biocompatible, bioreactive, and biodegradable; used in agriculture, food, environment, pharma, and health sectors.	[42,56]
Pectin	Present in plant cell walls within the middle lamella, occurring as calcium and magnesium salts	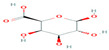 IUPAC:(2S,3R,4S,5R,6R)-3,4,5,6-tetrahydroxyoxane-2-carboxylic acid.	It is used as a gelling agent but can also act as a thickener, water binder, and stabilizer and affects texture and viscosity in the food industry.	[38]
Agar	Obtained from Gelidium amansii (Gelidaceae) and other red algae	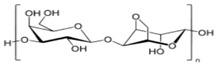 Agar consists of a mixture of agarose and agaropectin. It is composed of repeating alternate units of β-1,3-linked- D-galactose and α-1,4-linked 3,6 anhydro-L-galactose units.	Insoluble in cold water, dissolves readily in boiling water, and melts above 85 °C. Agar is used as a suspending agent, emulsifying agent, and gelling agent in tissue cultures and microbiology studies.	[43,57]
Alginate	Alginate is an anionic polymer and occurs naturally in the cell walls of brown algae (Phaeophyceae)	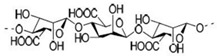 The alginates consist of 1,4-β-D-mannuronic acid (M) and 1,4 α-L-guluronic acid (G) monomers, with a homogeneous (poly-G, poly-M).	Acts as an antimicrobial agent to reduce the reproduction of Rhizoctonia (fungi) disease in potatoes and enhances the uptake of some nutrient elements and fertilizers.	[44,58]
β-cyclodextrin	From different starch sources such as potato, corn, wheat, rice, and tapioca	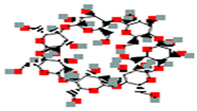 These are cyclic oligosaccharides commonly composed of six, seven, or eight D-glucose units (α-, β-, and γ-cyclodextrins, resp.) joined by α-(1,4) glycosidic bonds.	Form molecular inclusion complexes with many compounds and thus have applications in the fields of medicine, food, pharmaceuticals, and cosmetics.	[59]
Dextran	Dextran is a complex branched glucan (polysaccharide derived from the condensation of glucose)	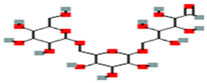 Branched poly-α-d-glucosides of microbial origin with glycosidic bonds predominantly C-1 → C-6.	Used as plasma volume expanders and anticoagulants. They are also commonly used in biological experimentation.	[60,61]
Lignin	Lignin is the heterophenolic polymer found in the plant cell wall, particularly wood and bark	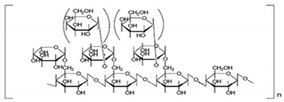 Lignin is composed of up to three different phenyl propane monomers, such as coniferyl alcohol, syringyl alcohol, and coumaryl alcohol units.	Lignin + sulphated lignosulfonates are used as dispersants, binders, complexing agents, and emulsifying agents. Barrier against attack by insects and fungi.	[38]
β-D-glucan	Found in a wide variety of cereal, plant, algae, bacteria, fungi, and yeast sources	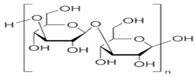 Linear homopolysaccharides composed of D-glucopyranosyl residues (Glc p) linked via a mixture of β-(1 → 3) and β-(1 → 4) linkages.	Its structural conformation and molecular weight make it useful in foods and biological entities.	[62]
Carboxymethylcellulose	Cellulose derivative formed by its reaction with alkali and chloroacetic acid	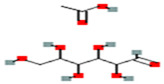 β-(1 → 4)-D-glucopyranose polymer chain of cellulose.	Low toxicity, biodegradable, and excellent film-forming ability. Strong network structure and hydrophilicity.	[63,64]
Polyhydroxybutyrate	Macromolecules synthesized by bacteria (Bacillus) under stress conditions	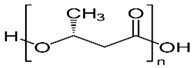 [4-Oxo-4-(4-oxobutan-2-yloxy)butan-2-yl] 3-hydroxybutanoate	Biodegradability, use of renewable resources, better physical properties and non-toxic.	[65]
Carrageenan	Extracted from different species of red seaweeds	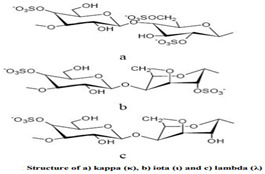 (β-(1,4)-D-galactose-α-(1,3)-D-galactose).	Types: kappa (κ), iota (ι), and lambda (λ); these are used in the food industry as gelling agents and thickeners. The iota carrageenan contains two sulfated groups per repeating unit.	[57,66]
Gum acacia/gum arabic	*Acacia senegal* and *Acacia seyal* tree exudate	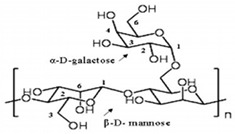 1,3-linked β-D-galactopyranosyl unit is present. L-arabinose, L-rhamnose, and D-glucuronic acid have also been identified as components of this polymer.	Ca; Na; K; P; and traces of Pb, Co, Cu, Zn, Ni, Cd, Cr, and Mn are the most common minerals detected. High capacity for polymerization.	[36,67]
Guar gum	From seeds of guar gum (*Cyamopsis* *Tetragonoloba*)	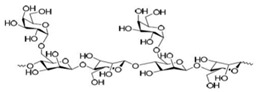 1 → 4-linked β-D-mannopyranose backbone with branch points from their 6 positions linked to α-D-galactose (i.e., 1 → 6-linked α-D-galactopyranose), every galactose residue between 1.5–2 mannose residues. Functional groups: CH_2_OH, OH, O, H.	Economical thickening and stabilizing agent; viscous pseudoplastic; high low-shear viscosity; shear thinning; less sensitive to ionic strength or pH; synergistic action with xanthan gum.	[43]
Cashew gum	Exudate of the cashew tree (*Anacardium occidentale* L.)	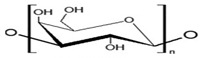 Cashew gum is built up of D-galactose, L-arabinose, D-galacturonic acid, and L-rhamnose (in traces). Functional groups: OH, O, COOH.	Application as a gelling agent, in polyelectrolyte complexes, as a viscosity enhancer, in controlled delivery systems, as a surfactant, as a drying aid agent, as a coating agent, and in microencapsulation.	[47,68]
Xanthan gum	Obtained by fermentation of Gram-negative bacteria, *Xanthomonas campestris*	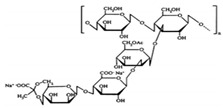 Five sugar residues: two glucose, two mannose, and one glucuronic acid. Functional groups: CH_2_OH, OH, O, COOH, H.	High viscosity even at low concentrations. Compatibility with heat, pH, shear, enzymes, and chemicals as well as variations in ionic strength.	[41,69]

**Table 2:** Biopolymeric nanoparticle-based carriers of fungicides used to target pathogenic fungi.

Fungicide	Nanoparticles	Crop	Targeted Pest	Toxicity or Soil Leaching	Reference
Pyraclostrobin	Chitosan–PLA graft copolymer	-	*C. gossypii* Southw	-	[86]
Imidazole (prochloraz)	Silica–alginate	-	*-*	-	[93]
Carbendazim and tebuconazole	Polymeric and solid lipid NPs	Bean seeds	-	Mouse fibroblast cells and soil sorption	[12]
Propiconazole	Chitosan	Wheat (*T. aestivium*)	Fusarium head blight by *F. graminearum*	-	[94]
Chitosan NPs	Chitosan	Rice	Rice blast by fungus *Pyricularia grisea*	-	[95]
Chitosan and its NPs	Chitosan	Wheat	Fusarium head blight by *Fusarium graminearum*	Maximum inhibition at 5000 ppm	[94]
Ketoconazole	Chitosan–gallen gum nanocomposite	-	*Aspergillus niger*	-	[96]
Pyraclostrobin	Chitosan and MSN	-	*Homopsis asparagi (Sacc*.)	-	[97]
Hexaconazole	Chitosan and tripolyphosphate (TPP)	-	*R. solani*	Vero cell lines	[98]
Mancozeb	Chitosan- gum acacia	Tomato	*A. alternata, S. lycopersici*	*-*	[99]
-	Cu–chitosan nanocomposite	-	*R. solani* and *S. rolfsii*	-	[100]
Cymbopogon martini essential oil *	Chitosan	Maize grains	*F. graminearum*	-	[101]
Copper-oxy-chloride	Chitosan copper oxide/zinc oxides	Chickpea (*Cicer arietinum)*	*F. oxysporum* f. sp. ciceri (FOC)	-	[9]
Metalaxyl	Chitosan	Millet	Downy mildew caused by *Sclerospora graminicol*	-	[102]
Chitosan NPs	Chitosan	-	*A. tenuis, A. flavus, F. graminearum, F. oxysporum, S. rolfsii*	Zearalenone production inhibited at 800 ppm	[103]
Spinosad	Chitosan–isoleucine	-	*Fusarium oxysporum*	Protection against photo-degradation	[90]
Clove essential oil *	Chitosan	-	*A. niger*	-	[104]
Chitosan and its NPs	Chitosan	Tomato (*Solanum lycopersicum*)	*C. gelosporidies, Phytophthora capsici, S. sclerotiorum, F. oxysporum, G. fujikuori*	-	[89]
Hexaconazole-dazomet	Chitosan	Palm	Basal stem rot disease by *G. boninense*	A 74.5% reduction in disease	[91]
Azoxystrobin, pyraclostrobin, tebuconazole, and boscalid	Lignin	*Vitis vinifera*	Grapevine trunk disease by *Phaeomoniella chlamydospora* and *Phaeoacremonium minimum*	Test fungi growth inhibition after 96 h in vitro	[92]
Mancozeb	Chitosan–carrageenan	Potato and tomato,	*A. solani, A. alternata, S. lycopersici, Sclerotinia* *Sclerotiorum*	Showed good antifungal efficacy in vitro and in vivo	[105]
Mancozeb	Chitosan–gum acacia	Potato	Early blight and stem rot (*Alternaria solani, Sclerotinia* *Sclerotiorum*)	Inhibition of 83.8 and 100%, respectively	[106]
Mancozeb	Chitosan	Tomato *(Solanum lycopersicum)*	Fusarium wilt	Inhibited the disease in vitro	[107]

* Oils/extracts with fungicidal activity that act at specific target sites but are not classed as fungicides.

**Table 3:** Biopolymeric nanoparticles tested as carriers of insecticides.

Insecticide	Nanoparticles	Target Pest	Reference
Imidacloprid	Chitosan/sodium alginate	-	[115]
Carbofuran	PVA–starch composite	-	[116]
Etofenprox	Chitosan	*Spodoptera litura*	[117]
Pyrifluquinazon	Chitosan	Peach aphid	[118]
Imidacloprid	Lignin	-	[119]
Azadirachtin *	Chitosan	-	[120]
Deltamethrin *	Chitosan-coated beeswax SLN	-	[121]
Azadirachtin *	Chitosan	Tobacco cutworm (*S. litura*)	[122]
Chlorpyrifos	Chitosan/PLA	-	[123]
Thiamethoxam	PLA/cellulose nanocrystal	Whiteflies	[124]
Imidacloprid	Sodium alginate	Leafhoppers (jassids)	[10]
Methomyl	Azidobenzaldehyde and carboxymethyl chitosan	Armyworm larvae	[109]
Pyridalyl	Sodium alginate	*Helicoverpa armigera* larvae	[110]
Chlorpyrifos	Sodium alginate	Nematode	[125]
Malathion and spinosad	chitosan/alginate/gelatin	*Culex pipiens* larvae	[126]
PONNEEM	Chitosan	Cotton bollworm larvae *(H. armigera*)	[127]
Cypermethrin	Chitosan and diamotite	Corn borers	[128]
Emamectin benzoate	Ethyl cellulose	*Plutella xylostella*	[129]
Fungal biopesticidal agent *Nomuraea rileyi*	Chitosan	*Spodoptera litura*	[130]
Azadirachtin *	Zinc oxide and chitosan	Groundnut bruchid (*C. serratus*)	[112]
Carvacrol */linalool	Chitosan	Mite (*T. urticae*)	[50]
Geraniol *	Chitosan/gum arabic	Whitefly (*B. tabaei*)	[111]
*S. hortensis* L. essential oil *	Chitosan/TPP	Mite (*T. urticae*)	[131]
Nicotine *	Chitosan/TPP	House fly (*M. domestica*)	[132]
Spinosad and permethrin	Chitosan	*Drosophila melanogaster*	[17]
Emamectin benzoate	Carboxymethyl chitosan	*Mythimna separate*	[113]
Thiamethoxam	Cellulose	*Phenacoccus solenopsis*	[20]
Pyridalyl	Sodium alginate	*Helicoverpa armigera*	[133]

* Oils/extracts with insecticidal activity that act at specific target sites but are not classed as pesticides.

**Table 4:** Biopolymeric nanoparticles assessed as carriers of herbicides.

Herbicide	Nanoparticles	Target Crop	Toxicity or Soil Leaching	Reference
Paraquat	Alginate/chitosan	-	Lara and Carvoeiro soil sorption	[134]
Atrazine	Poly(caprolactone) coated in chitosan	-	-	[136]
Paraquat	Chitosan/tripolyphosphate	Mustard (*Brassica* sp.) and maize	Chinese hamster ovary cells, *A. cepa*, soil sorption	[135]
Diuron	Chitosan	Maize	*E. crus-galli*	[137]
Imazapic and imazapyr	Alginate/chitosan and chitosan/TPP	Black-jack (*B. pilosa)*	Chinese hamster ovary cells, *A. cepa,* soil biota	[138]
Clomazone	Chitosan/alginate	-	Bullfrog tadpole (*L. catesbeianus*) livers	[139]
Atrazine	Chitosan/tripolyphosphate	-	*C. elegans*	[140]
Metsulfuron methyl	Pectin nanocapsules	*Chenopodium album*	Vero cell lines	[47]
2,4–dichloro phenoxy acetate	Starch and sodium alginate	-	-	[141]
Glyphosate	Diatomite/Fe_3_O_4_ with chitosan	-	-	[128]
Atrazine	Poly(lactic-co-glycolic acid)	Potato plant	-	[142]
Atrazine	N-hexanoyl-O-glycol chitosan	-	-	[143]
Atrazine	poly(lactic-co-glycolic acid)	*-*	-	[144]
Tebuthiuron	Sodium alginate	Cucumber plants	The leaching of commercial herbicide in soil was 40–50 cm deep, while it was 20–30 cm for nanoformulations	[145]
Clomazone	Polymeric nanoparticles	*-*	Higher toxicity in tadpoles (*L. catesbeianus*) in the form of lipidosis and macrophage aggregation by the herbicide	[146]

**Table 5:** Polymeric nanoparticles developed as carriers of nanonematicides.

Nanocomposite/Polymer	Disease/Plant	Target Nematodes	Reference
(N-methyl-N-cis-styrylcinnamamide)	Pine wilt disease	*Bursaphelenehus xylophilus* and *Meloidogyne incognita*	[150]
Starch/silver	Bermudagrass disease	*M. incognita*	[151]
Silver/titanium oxide NPs	Root-knot disease in tomato	*M. incognita*	[152]
Poly(lactic-co-glycolic acid)-b-poly(ethylene glycol) (PLGA-b-PEG)	Southern root-knot disease	*Meloidogyne incognita*	[153]

**Table 6:** Biopolymeric nanoparticles studied as carriers of nanomolluscicides.

Nanocomposite	Disease/Plant/Toxicity	Target Mollusca	Notes	Reference
Nisin-curcumin-polylactic acid	Reduction in egg-laying capacity	*Biomphalaria pfeifferi*	NPs prepared by the double-emulsion/diffusion–evaporation method	[154]
Nisin-curcumin-polylactic acid	Schistosomiasis and fascioliasis	*Biomphalaria pfeifferi, Bulinus globosus,* and *Lymnaea natalensis*	NPs prepared by the double-emulsion/diffusion–evaporation method	[155]

**Table 7:** Polymeric nanoparticles tested as carriers of nanomiticides.

Nanocomposite/Polymer	Disease/Plant/Toxicity	Target	Notes	Reference
Poly(ethyleneimine) and silica	Fipronil, an insecticide, was encapsulated	Termite colonies of *Coptotermes lacteus*	PEI-coated silica nanocapsules formulated with α-cellulose as bait toxicants against nine termite colonies	[156]

**Table 8:** Antimicrobial activity of some biopolymeric nanocomposites.

Nanoparticle Matrices	Active Agent	Pathogens	Disease	Characteristics	Reference
Ag/chitosan nanoformulations	-	*Alternaria* and *Rhizoctonia* species	Seed-borne diseases	NPs exhibited inhibition against *Aspergillus > Alternaria* > *Rhizoctonia* species	[162]
Chitosan nanoparticles LMW/HMW	Chitosan NPs of different molecular weights	*C. albicans* *F. solani* *Aspergillus niger*	-	Except for HMW, all showed high antimicrobial activity against *Aspergillus niger*	[157]
Cu–chitosan nanoparticles	-	*Fusarium greminaerum*	-	Inhibited the mycelial growth	[163]
Chitosan–Cu nanoparticles	Different concentrations	*A. alternata, M. phaseolina, R. solani,* and *F. oxysporum*	-	Chitosan–Cu NPs inhibited the mycelial growth of these fungi	[164]
Chitosan–silver nanoparticle composite	-	*Colletotrichum gloeosporioides*	Anthracnose mango	Composite inhibited conidial germination of *C. gloeosporioides* and reduced the anthracnose incidence in mango	[158]
Chitosan–copper nanocomposites (Cu/Ch) and chitosan–zinc nanocomposites	-	*A. alternata, R. solani* and *A. flavus*	-	All the fungi showed maximum activity	[165]
β-D-glucan nanoparticles	-	*Pythium aphanidermatum*	Rhizome rot disease of turmeric	About 77% protection against rhizome rot disease was found in NP-treated plants	Anusuya [166]
Chitosan–g-acrylonitrile silver nanocomposite	-	*Aspergillus niger*	-	18 mm inhibition zone was observed against *Aspergillus niger*	[167]
Chitosan–Cu NPs	-	*A. solani, F. oxysporum*	Tomato	NPs showed good antimicrobial activity	[168]
Cu–chitosan and Zn chitosan NPs	-	*Rhizoctonia solani* and *Trichoderma logibrachiatum*	Cotton seedlings damping-off disease	Cu–chitosan nanocomposite showed the highest antifungal activity against *R. solani*	[169]
Chitosan–Ag nanocomposite and chitosan NPs	-	*Fusarium oxysporum*	*-*	Chitosan–Ag nanocomposite showed a significantly higher radial growth inhibition than chitosan NPs for all the tested concentrations	[170]
Cu–chitosan nanocomposite	-	*R. solani* and *S. rolfsii*	-	Inhibited the growth of both *S. rolfsii* and *R. solani*; AG-4 was observed solvent; a loss of the cytoplasm content and destruction in the hyphae was confirmed	[100]
Starch nanoparticles	Phenyl and cyclohexyl groups	*Phomopsis asparagi, Colletotrichum lagenarium,* and *Fusarium oxysporum*	Watermelon fusarium	Nanoformulation showed good antifungal efficacy	[171]
Cu–chitosan	-	*Pyricularia grisea*	Finger millet	75% control of the disease	[22]
Ch-CuO and Ch-ZnO nanocomposites	Copperoxy-chloride (CuOCl)	*Fusarium oxysporum* f. sp. *ciceri* (FOC)	Fusarium wilt disease of chickpea	All NPs showed good antifungal efficacy and were found to promote the growth of chickpea plants	[9]
Oligochitosan–silica/carboxymethyl cellulose	-	*Phytophthora infestans*	-	800 mg/L was the lowest concentration that inhibited fungal growth	[161]

**Table 9:** Research on controlled-release matrices of active ingredients encapsulated in biopolymers and their use in agriculture.

Matrices	Synthesis Method	Active Ingredient	Characteristics	Reference(s)
Chitosan gel beads	Cross-linking	Atrazine (herbicide) and urea	Prolonged release period of atrazine to 7 months and urea to 180 h	[178]
Alginate/bentonite	-	Atrazine (herbicide)	No presence of herbicide in the leachate according to the controlled-release soil column model	[33]
Chitosan microcapsules	Precipitation	3-Hydroxy-5- methylisoxazole (herbicide)	Sustained release of active material in water for 80–160 h	[179]
Alginate–chitosan microcrystals	Self-assembly	Imidacloprid (insecticide)	Photo-degradable insecticide; showed toxicity against *Martianusdermestoides* adults	[115]
Starch and poly(methacrylic acid)	Cross-linking	Thiram (fungicide)	A non-Fickian diffusion mechanism was observed	[180]
Lignin	-	Isoproturon, imidacloprid, and cyromazine (pesticides)	All three pesticides showed diminished release compared to commercial formulations	[181]
Starch and alginate	Cross-linking (CaCl_2_ as cross-linker)	Thiram (fungicide)	The entrapment efficacy of thiram was more than 90%, and a case II diffusion mechanism was observed	[182]
N-(octadecanol-1- glycidyl ether)-Osulfate chitosan (NOSCS) micelle	Reverse micelle	Rotenone (insecticide)	Useful as a prospective carrier for controlled-released agrochemicals	[183]
Chitosan microspheres	Coacervation–cross-linking	Brassinosteroids (hormones)	Controlled delivery of Brassinosteroids with activity as agrochemicals	[184]
Chitosan microspheres	Cross-linking	Paraquat (herbicide)	Sustained release of active material in water for 8 h	[134]
Lignin and ethylcellulose	-	Chloridazone (herbicide)	The rates of release of chloridazone were lower for the new formulations compared to the commercial product	[185]
Poly hydroxybutyrate (PHB) and poly hydroxybutyrate valerate (PHBV)	Emulsion/solvent evaporation	Ametryn (herbicides)	Slow and sustained release (reduced to 75% and 80%) as compared to pure ametryn	[186]
Chitosan–silver microbeads	Cross-linking	Silver nanoparticles	Pesticide removal for extended periods	[187]
Chitosan nanoparticles + chitosan	-	Dichlorprop (herbicide)	Enhanced toxicity to fresh water green algae and slow release of dichlorprop	[188]
Chitosan–PVA hydrogel	Cross-linking	Silver nanoparticles	Small size (13 nm); exhibited good antibacterial activity	[189]
Alginate-reinforced chitosan and starch beads	Cross-linking	Imazaquin (herbicide)	Porous spherical beads of 2.31 mm size; sustained slow release of active material	[190]
Cu–chitosan nanoparticles	Ionic gelation	CuSO_4_	Improved antifungal activity against *Alternaria alternate, Macrophomina phaseolina,* and *Rhizoctonia solani*	[164]
Au–carrageenan	Blending the polymer matrix	Methylene blue as model drug	After 5 h the drug release from the composites slowed down	[15]
Beauvericin–chitosan nanoparticles	Ionic gelation	Beauvericin (pesticidal cyclodepsipeptide)	Enhanced pesticidal activity against groundnut defoliator	[191]
Silica–alginate	Polymerization and complexation	-	Release rates reportedly increased from 69.2% to 82.1% on the 60th day	[93]
Alginate–chitosan nanocapsules	Polyelectrolyte complexation	Acetamiprid	Controlled-release pattern with maximum release at pH 10	[11]
Chitosan–xanthan gum	Ionotropic gelation method	Thiabendazole	Nanoformulation showed better results as compared to pure thiabendazole	[192]
Chitosan and tripolyphosphate (TPP)	Ionotropic gelation method	Hexaconazole	Hexaconazole release was fastest at pH 4.0 compared to pH 7.0 and 10	[96]
Alginate/chitosan and chitosan/tripolyphosphate	Polyelectrolyte complexation	Imazapic and imazapyr	Release percentages of 59 and 9% were obtained for imazapic and imazapyr, respectively, after 300 min	[138]
Chitosan and pectin	Ionic interaction	Carbendazim	Sustained release	[88]
Chitosan and β-Cyclodextrin-epichlorohydrin	Polyelectrolyte complexation	Carbendazim	Retarded the release rate of fungicide	[193]
Cu/Zn-loaded PVA–starch nanocomposite	Blending the polymer matrix	Micronutrient	The release of micronutrients and Cu/Zn from polymeric nanocomposite was controlled	[194]
N-hexanoyl-O-glycol chitosan	-	Atrazine	The micelle enhanced the water solubility of atrazine three-fold	[143]
Chitosan	Ionic gelation method	Hexaconazole	Sustained release of 99.91% with a prolonged time of 86 h	[81]
Alginate–cellulose	Ionotropic gelation method	Imazethapyr (herbicide)	The time taken for 50% release of herbicide was increased from 11.30 days to 43.73 days using nanoformulations	[177]
Chitosan	Blending the polymer matrix	Glyphosate	High-molecular-weight chitosan caused a slower release of the herbicide than low-molecular-weight chitosan nanoformulations	[83]
Chitosan	Ionic gelation method	Spinosad and permethrin	30% spinosad and 75% permethrin were released within 5 h	[17]
Alginate–polyacrylamide		λ-cyhalothrin	The hydrogel showed the lowest cumulative release percentage of 6.68% over 87 h	[82]
Copper chitosan	Chemical reduction	Dicamba dimethylamine	The release of dicamba in aqueous medium and agricultural soils was successful	[176]
Chitosan carrageenan	Ionic gelation	Mancozeb	Commercial mancozeb was released within 2 h, while nanoform took 10 h	[105]

## Data Availability

Not applicable.
